# La ténorraphie percutanée dans les ruptures fraîches du tendon calcanéen: à propos de 67 cas

**DOI:** 10.11604/pamj.2014.17.287.3278

**Published:** 2014-04-15

**Authors:** Aman Bessam, Zouhir Ameziane Hassani, Mohamed Kharmaz, Alain Steinmetz

**Affiliations:** 1 Hôpital Avicenne, Service Traumatologie Orthopédie de Rabat, Maroc; 2 Centre hospitalier de Mulhouse, France

**Keywords:** Ténorraphie percutanée, rupture fraîche, tendon calcanéen, sport, Percutaneous tenorraphy in fresh ruptures of the Achilles tendon: report of 67 cases, Fresh rupture, Achilles tendon, sport

## Abstract

La rupture du tendon calcanéen est une des lésions les plus fréquentes en pathologie du sport, les deux options thérapeutiques classiques sont le traitement orthopédique et la chirurgie à ciel ouvert. Dans le but de minimiser ces complications, des techniques mini-invasives de ténorraphie percutanée ont été proposées dont les résultats sont encourageants. Il s'agit d'une étude rétrospective d'une série de cas de rupture sous cutanée du tendon d'Achille s’étalant de Avril 2005 au Juin 2012 concernant 67 patients; 11 femmes et 56 hommes avec un âge moyen de 41 ans. La cause principale était un accident de sport dans 45 cas. Le diagnostic était évident à l'examen chez tous les patients. Dans deux cas il s'agissait d'une rerupture survenant à 1 et 5 ans d'un traitement orthopédique. Dans un cas il s'agissait d'une rerupture survenant après une ténorraphie percutanée. Dans deux cas il s'agissait d'une rupture sur tendinopathie chronique. Tous les patients avaient bénéficié d'une radio de la cheville qui avait montré une horizontalisation du calcanéum chez 5 patients et surtout elle n'avait pas montré de fracture associée, alors que 15 patients avaient bénéficié d'une échographie qui a confirmé le diagnostic. Tous les patients avaient été opérés dans un délai de moins de 48 heures Une ténorraphie percutanée a été pratiqué chez tous les patients. Le recul moyen est de 43 mois, trois patients ont été perdus de vue Nous avons noté une reprise des activités professionnelle effective en moyen 90 jours après l'intervention et celle des activités sportive à 6 mois en moyenne L’état cutané local a été jugée bon dans 63 cas. Par ailleurs les complications ont été marquées par un seul cas d'infection ayant nécessité une reprise chirurgical a été noté mise à plat, trois cas de rerupture repris par suture à ciel ouvert, un cas d'algodystrophie et un cas de tendinopathie Il n'y a eu aucune complication thromboembolique ni neurologique. Il n'existe pas de réel consensus concernant la prise en charge des ruptures du tendon calcanéen. Ainsi, La ténorraphie percutanée du tendon calcanéen est une méthode simple rapide et efficace, elle allie les avantages de la chirurgie à ciel ouvert en termes de pourcentage de rerupture et les avantages du traitement fonctionnel en rapport avec un faible risque infectieux.

## Introduction

La rupture du tendon calcanéen est une des lésions les plus fréquentes en pathologie du sport; Si le diagnostic de la lésion est aisé, son traitement prête aujourd'hui encore à controverse. Les deux méthodes thérapeutiques principales sont le traitement conservateur et le traitement chirurgical à ciel ouvert. Le traitement conservateur consiste en une immobilisation en équin pendant 12 semaines mais comporte un risque de rerupture. Le traitement chirurgical comporte des risques septiques et de cicatrisation cutanée. Dans le but de minimiser ces complications, des techniques mini-invasives de ténorraphie percutanée ont été proposées dont les résultats sont encourageants.

## Méthodes

De avril 2005 au juin 2012 une série rétrospective et continue des ténorraphie percutanées du tendon calcanéen a été réalisée. Il s'agissait de 67 patients 11 femmes et 56 hommes âgés de 41 ans (24-70). Le mécanisme avait été un accident sportif dans 45 cas (football:20, tennis:cinq, basketball:trois, handball:deux, non précisé:15); un traumatisme indirect (chute, faux mouvement)dans 14 cas; traumatisme direct dans huit cas. Parmi les 45 ruptures survenues au cours d'une activité sportive; neuf sont survenus alors que le patient était considéré comme sédentaire, et douze cas comme actif. Au total 33 patients (49%) pratiquaient une activité sportive régulière au moment de la rupture; le niveau sportif de ces patients évalué selon la cotation Arpège Clas, était le suivant: Cinq compétiteurs et 30 sportifs de loisirs. Dans deux cas il s'agissait d'une rerupture survenant à 1 et 5 ans d'un traitement orthopédique. Dans un cas il s'agissait d'une rerupture survenant après une ténorraphie percutanée. Dans deux cas il s'agissait d'une rupture sur tendinopathie chronique. Toutes les ruptures étaient évidentes à l′examen [1–3]. Tous les patients ont bénéficié d′une radio de la cheville qui a montré une horizontalisation du calcanéum chez 5 patients et surtout elle n′a pas montré de fracture associée, alors que 15 patients ont bénéficié d'une échographie qui a confirmé le diagnostic [4–6]. Dans 57 (85%) cas, la rupture siégeaient en plein corps du tendon, et dans 10 cas (15%), la rupture siégeait à la jonction musculotendineuse. Tous les patients ont été opérés dans un délai inférieur à 48 h. La duré d′hospitalisation: entre 3 et 5 jours avec une durée moyenne de 3,8 jours.

**Figure 1:**
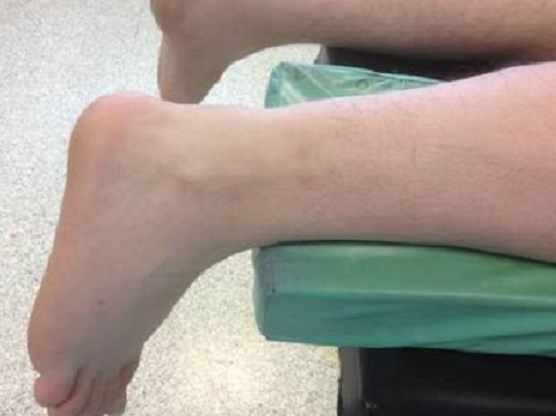
Installation opératoire

Technique opératoire: Le patient est installé en décubitus ventral, sous anesthésie loco- régionale, sans garrot pneumatique. Un petit appui est placé sous le cou de pied afin de faciliter la mise en position en équin (Figure 1).

**Figure 2:**
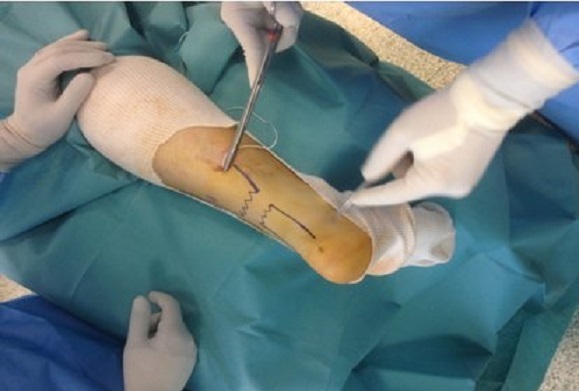
Introduction de l'aiguille

**Figure 3:**
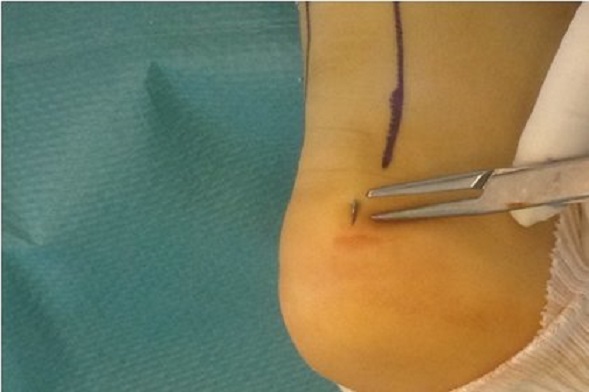
Sortie de l'aiguille

La rupture est repérée au palper. Une moucheture est pratiquée à environ 4 à 5 cm en amont de la partie proximale de la rupture. L′aiguille est alors modelée en fonction de la distance existant entre le point d′introduction et le point choisi de sortie. Elle est introduite dans l′axe du tendon du haut vers le bas. La progression et la direction de la pointe pouvant être contrôlées en permanence par le palper. Le fragment distal, est aisément transfixié dans son épaisseur sur environ 4 à 5 cm selon le niveau de la rupture (Figure 2). L′aiguille étant à peu près au niveau de l′insertion calcanéenne, on la fait émerger dans la fossette rétro-malléolaire interne ou externe selon le premier point d′entrée choisi. Il est procédé de la même façon avec une deuxième aiguille montée qui est placée de façon symétrique et opposée à la première (Figure 3). Le pied étant maintenu en équinisme maximum, les fils sont mis en tension ce qui assure un télescopage des fibres du tendon rompu à l′intérieur de la gaine. Le blocage des fils à la sortie est réalisé par un plomb perforé appuyé sur la rondelle de plastique convexe. Les mouchetures proximales sont laissées ouvertes autour du fil serti, lui-même libre car il permettra l′ablation ultérieure simple par traction. Une botte plâtrée est réalisée maintenant l’équin obtenu lors de la suture et remplacée à 3 semaines par une deuxième botte avec une immobilisation proche de l'angle droit pour une nouvelle durée de trois semaines. l'appui est autorisé dès la 3ème semaine [7]. Tous nos patients ont bénéficié d'une rachi anesthésie.

Les patients ont été revus avec une fiche d’évaluation comprenant: l’état cutané; le délai de reprise de l'appui, du travail et du sport; l'amyotrophie du mollet; la comparaison de la largeur du tendon opéré avec le tendon sain; l'appui monopodal; le saut monopodal sur la pointe des pieds, la marche sur la pointe des pieds; les amplitudes articulaires en flexion dorsal et plantaire, la satisfaction du patient.

## Résultats

Le recul moyen est de 43 mois (6 - 89). Trois patients ont été perdus de vue.

**Figure 4:**
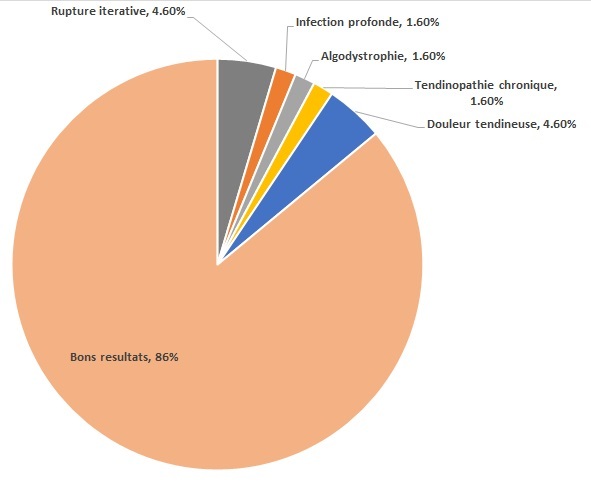
Résultats

**Complications:** Un cas de complications infectieuses nécessitant une reprise chirurgicale pour mise à plat. Trois cas ont eu une rerepture repris par une suture chirurgicale à ciel ouvert. Trois patients ont présenté une douleur post opératoire, un cas a eu une algodystrophie, et un cas a eu une tendinopathie nécessitant une reprise chirurgicale pour peignage du tendon à 1 an de la suture.il n'y a pas eu de complication thromboemboliques ni neurologique par atteinte du nerf sural (Figure 4).

**Reprise des activités professionnelles:** A été effective en moyenne 90 jours après l'intervention.

**Reprise des activités sportives:** A été effectuée en moyenne à 6 mois (3-12) après le traumatisme.les cinq sportifs de compétition ont repris leur activité sportive à 6 mois au même niveau.la reprise des activités sportives a été possible chez 25 sportifs de loisir(84%).parmi ces patients 20 (67%)ont repris au même niveau de performance.une diminution du niveau a été noté chez cinq patients (17%). cette baisse du niveau était associée dans trois cas à une douleur post opératoire et dans 2 cas à une crainte de rerupture.

**Bilan clinique:** L’état cutané local était bon dans 63 cas (98%); 17 cas (26%) présentaient une augmentation du périmètre de la cheville. une augmentation de la largeur du tendon opéré par rapport au tendon sain a été remarqué chez 19 cas (29%). Une amyotrophie du mollet homolatéral a été remarqué chez 3 patients est toujours restée inférieure à 2cm (5%). l'appui monopodal était possible chez tous les patients. Le saut monopodal n'a pas été possible que dans 37 cas. la marche sur la pointe des 2 pieds n'a été possible que dans 29 cas. Un déficit de flexion dorsale de cheville de 5° a été noté chez deux patients. Trois patients gardent une douleur tendineuse.les résultats subjectifs des patients ont été les suivants: 22 très satisfaits; 41 satisfaits, et 1 mauvais.

## Discussion

La rupture du tendon d′Achille est une lésion de l'adulte d’âge moyen; elle est beaucoup plus fréquente chez l′homme par rapport à la femme cela s′expliquant par la fréquence de l′activité sportive chez les hommes. La majorité des patients de notre série ont eu une rupture lors d′une activité sportive; la rupture se produit plus chez le sportif e ce qui a été rapporté dans toutes les séries de la littérature; citant celle de Rouvillain [8] où 68% sont des sportifs, et celle de Lecestre [9] où 67% sont des sportifs. La prédominance des atteintes chez les amateurs est notée également dans toutes les séries [8–10] peut être expliquée par la mauvaise éducation (absence d’échauffement...), et par la reprise aiguë de l′activité sportive après un arrêt.

Dans notre série l'interrogatoire et l'examen clinique étaient faciles et suffisants pour poser le diagnostic, ce qui coïncide avec les données de la littérature où les examens complémentaires ne sont fait que pour éliminer d'autres lésions (radio standard) ou à titre complémentaire (échographie et IRM) [11]. Tous nos patients ont bénéficié d'une ténorraphie percutanée. Cette méthode utilisant le Ténolig^®^ [12] assure des résultats au moins équivalents à la chirurgie sans en avoir les inconvénients parce que c′est un geste simple rapide ne nécessitant qu′une hospitalisation de courte durée et ne provoque pas de complications cutanées significatives avec un risque de rupture itérative à peine supérieur à celui de la chirurgie à ciel ouvert [13], en plus de la supériorité du traitement percutané sur le plan esthétique. Cependant malgré les résultats excellents obtenus dans le traitement par fixateurs externes dans quelques publications, il demeure rarement utilisé car il est difficile et nécessite des soins intensifs.

A propos du traitement orthopédique, il est encore considéré par beaucoup d′auteurs comme efficace, et il donne des résultats objectifs en tout point comparables à ceux des autres méthodes avec bien des avantages: absence d′hospitalisation et d′anesthésie, aucun trouble lié à la cicatrice et aucune adhérence cutanée. Les risques sont: l′allongement du tendon, la longue durée de traitement, et la rupture itérative. Pour comparer entre les différentes méthodes nous allons se baser sur l’étude d'Andrej Cretnik [14] concernant 105 patients traités par chirurgie à ciel ouvert et 132 patients traités par Ténorraphie percutanée en proposant la méthode percutanée comme une méthode de choix car elle apporte des résultats fonctionnels comparables à la réparation ouverte, avec un taux sensiblement inférieur de complications. Justin Lim [15] a conclu que la réparation percutanée est recommandée sur la base du bas taux de complications et l′aspect cosmétique amélioré.

## Conclusion

Il n'existe pas de réel consensus concernant la prise en charge des ruptures du tendon calcanéen. Ainsi, La ténorraphie percutanée du tendon calcanéen est une méthode simple rapide et efficace, elle allie les avantages de la chirurgie à ciel ouvert en termes de pourcentage de rerupture et les avantages du traitement fonctionnel en rapport avec un faible risque infectieux. Le traitement orthopédique impose une immobilisation prolongée de la cheville (supérieure ou égale à dix semaines) avec des délais de récupération longs et souvent partiels de la force du triceps Les traitements chirurgicaux sont plus certains d'obtenir un contact tendineux favorable à une cicatrisation solide, mais imposent une hospitalisation et des complications cutanées et parfois infectieuses graves. La ténorraphie percutanée peut apparaître comme le compromis idéal entre traitement chirurgical et conservateur, avec des résultats aux tests d’évaluation de la force musculaire les plus performants, qui en fait la technique de choix pour les patients sportifs et motivés. C'est à partir de ces raisons qu'on opte dans notre service pour la ténorraphie percutanée qui donnait de bons résultats sur le plan fonctionnel et ne fait courir le risque que de rares complications.
